# A12 EXAMINING THE ASSOCIATIONS BETWEEN PHYSICIAN OR PATIENT GENDER WITH REFERRAL PATTERNS IN GASTROENTEROLOGY FOR NON-SCREENING INDICATIONS

**DOI:** 10.1093/jcag/gwac036.012

**Published:** 2023-03-07

**Authors:** G Wang, A Boblitz, A Altaf, X Wang, E Benchimol, L Targownik

**Affiliations:** 1 Medicine, University of Toronto; 2 Life Stage Research Program, ICES; 3 Pediatric Gastroenterology, Hospital for Sick Children; 4 Gastroenterology, Mount Sinai Hospital, Toronto, Canada

## Abstract

**Background:**

Gender biases in referral may impact patient care, and may perpetuate gender-based pay inequities. Referral patterns of male and female patients to male and female gastroenterologists (GIs) have not been previously characterized.

**Purpose:**

We aimed to determine the extent to which female patients referred for consultation to gastroenterologists were preferentially channeled to female practitioners, and to further assess how gender-based referral channeling has changed over time.

**Method:**

We used data from IC/ES Ontario to identify all residents of Ontario, Canada who had had a new consultation with an Ontario gastroenterologist in an ambulatory setting between Jan 1, 2002, to Dec 31, 2019, with time subdivided into early (2002-2007), mid (2008-2013), and late (2014-2019) periods. New consults were defined as any GI consultation where there had been no ambulatory visit with a different GI in the two years prior. The primary outcome was the difference in the proportion of female patients seen by male GIs vs female GIs. Descriptive statistics were used to compare patient and GI characteristics. Continuous variables were analyzed by the t-test, with *p*<0.05 suggestive of statistical significance. Odds ratios and their 95% CIs for the association of referral to a female GI and being a female patient were calculated.

**Result(s):**

From 2002 to 2019, the proportion of female gastroenterologists in Ontario increased from 15% (15/100) to 27% (78/292). During this 18-year period, female GIs saw a total of 17% of all consultations. Male GIs saw a greater number of consultations per year, though the gap closed over the period of observation. Specifically, each female GI in 2014-19 saw, on average, 776 patients (±41.8) compared to 578 (±59.2) in 2002-2007 (*p*<0.005) – a 34.5% increase; each male GI in 2014-2019 saw, on average, 905 patients (±7.2) compared to 824 (±25.0) in 2002-2007 (*p*<0.005) – a 9.8% increase.

Female patients made up 56.7% of the total consultations over 2002-2019. There was evidence of channeling of female patients to female providers; in the early period, 72.4% of consults seen by female GIs were female, compared to only 56.8% of consults seen by male GIs (OR 2.07, 95% CI [1.98, 2.17]. By the late era (2014-2019), 64.1% of consults seen by female GIs were for female patients, compared to 53.3% for male GIs (OR 1.62, 95% CI [1.59, 1.66].

**Image:**

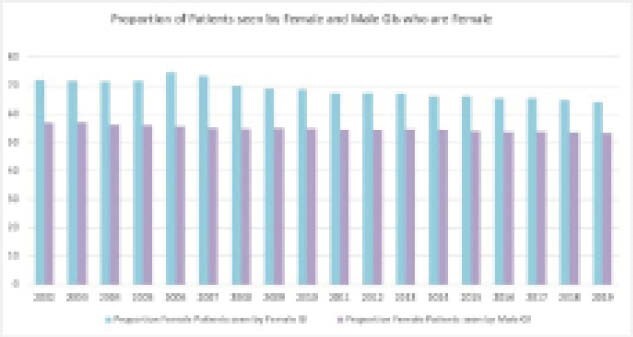

**Conclusion(s):**

There has been a significant increase in the number of female GIs in Ontario in recent years, and female GIs are seeing significantly greater patient volumes in the later eras in comparison to earlier eras. Female GIs receive a higher proportion of consultations for female patients, though this proportion is declining over time. The extent to which this gender-based referral channelling influences patient care, patient outcomes or influences the gender-based provider pay gap requires further exploration.

**Please acknowledge all funding agencies by checking the applicable boxes below:**

Other

**Please indicate your source of funding;:**

IMAGINE-SPOR

**Disclosure of Interest:**

None Declared

